# Long-Term Surgical Outcome of Class A and B Tympanomastoid Paragangliomas

**DOI:** 10.3390/cancers16081466

**Published:** 2024-04-11

**Authors:** Melcol Hailu Yilala, Giuseppe Fancello, Virginia Fancello, Lorenzo Lauda, Mario Sanna

**Affiliations:** 1 Department of Otology and Skull Base Surgery, Gruppo Otologico, 29121 Piacenza, Italy; 2 Department of Otorhinolaryngology, College of Health Sciences, Addis Ababa University, Addis Ababa P.O. Box 1176, Ethiopia; 3 Department of Otorhinolaryngology, Careggi University Hospital, 50134 Florence, Italy

**Keywords:** paraganglioma, glomus tympanicum, tympanomastoid glomus tumor, middle ear

## Abstract

**Simple Summary:**

Tympanomastoid paragangliomas are tumors arising from the middle ear an air-filled space located deep to the ear drum. These tumors rarely spread to other parts of our body. Patients who have these tumors usually experience ringing noise that is synchronous with the heartbeat in the affected ear with or without hearing impairment. During clinic visits, the ear examination usually shows a red mass behind the ear drum and decreased hearing levels. The mass is visible during CT and MRI scans. The main goal of treatment is complete surgical removal with preservation of hearing and facial function. The choice of surgical approach, however, depends on the size and extent of the tumor. Smaller tumors can be removed through the external ear canal. Whereas, larger tumors need surgical removal that requires a skin incision behind the ear to go to the external and middle parts of the ear. The objective of this study is to evaluate the usual presenting symptoms, clinical findings, imaging characteristics, the outcome of surgical management, and treatment-related complications including tumor recurrence. The results showed the mean age of presentation to be 54 and females were six times more likely to be affected than males. The most frequent symptoms were tinnitus and hearing loss while on office ear examination all patients had a red mass behind the ear drum. A High-resolution CT scan is the initial preferred modality of investigation that is important to determine the size and extent of the tumor. Depending on the size, 45% of our cases had tumors confined to the middle part of the ear whereas the rest 55% had bigger tumors with growth to the air-filled spaces around the middle ear and erosion of the bone that houses the carotid artery. All smaller tumors were removed through the external canal whereas larger tumors were removed by accessing the middle and external part of the ear from behind the auricle. Results after surgery showed excellent hearing and facial movement function. Complete tumor removal was achieved in 97% of the cases with 3% of recurrence after surgical removal. The most common complication after surgery was a permanent hole in the ear drum. Surgical treatment remains the preferred treatment modality with the benefits of complete disease removal and a lower rate of recurrence and complication.

**Abstract:**

Objective: To analyze the long-term facial function as well as overall postoperative condition in surgically treated tympanomastoid PGL patients. Study Design: Retrospective study. Method: The medical records of patients with surgically managed class A and B tympanomastoid PGLs between 1983 and 2023 were thoroughly evaluated. Result: Our center has treated a total of 213 cases of tympanomastoid PGL surgically. The mean age of patients was 54, and the male-to-female ratio was 1:6. The most common symptoms at presentation were hearing loss (80%), pulsatile tinnitus (77%), and vertigo (15%). According to the modified Fisch classification, 45% of the cases were classified as class A (A1 and A2), while 55% were classified as class B (B1, B2, and B3). All class A and most class B1 and B2 tumors were removed either with transcanal or retroauricular-transcanal approaches. However, more advanced class B3 lesions were removed with subtotal petrosectomy (SP) along with middle ear obliteration. Facial nerve outcome was excellent in all class A and B cases, while chances of postoperative paresis slightly increased with the size and extent of the tumor (*p* < 0.05). The hearing outcome is excellent for class A1, A2, B1, and B2 tumors, whereas more advanced class B3 cases have a loss of air conduction (AC) and increased bone conduction (BC) threshold (*p* < 0.05). Complete surgical removal was achieved in 97% of our cases. The most common late complication was permanent TM perforation (7%), and the recurrence rate was 3%. Conclusions: Tympanomastoid PGL represents the most common neoplasm of the middle ear space. The most frequent presenting symptoms include pulsatile tinnitus and hearing loss, whereas the presence of retrotympanic mass was evident in all cases at the time of initial otoscopic evaluation. Proper documentation of facial function and audiometric evaluation are crucial elements of preoperative workup. The most preferred preoperative radiologic examination is high-resolution computer tomography (HRCT), whereas magnetic resonance imaging (MRI) with or without gadolinium enhancement is reserved for cases with a dilemma of carotid artery or jugular bulb involvement. The main goal of tympanomastoid PGL treatment is complete disease removal with preservation of hearing and facial functions. Surgical treatment remains the preferred treatment modality with the benefits of complete disease removal, lower rate of recurrence and complication, and acceptable postoperative hearing level. Here, we present our 40 years of experience, which, to the very best of our knowledge, is the largest series of tympanomastoid PGL in the English literature.

## 1. Introduction

Head and neck PGLs are rare tumors of neuroendocrine origin that belong to paraganglioma origin [1,2]. Temporal bone PGLs are rare locally aggressive neoplasms of the temporal bone. These lesions include tympanomastoid and tympanojugular PGLs, which arise from the tympanic plexus area and the adventitia of the jugular bulb, respectively [3,4,5]. 

The presence of glomus tissue in the middle ear was first identified in 1941 by Gulid [5]. Subsequently, in 1945, Rosenwasser described the vascular supply of middle ear glomus tissues [6]. Tympanomastoid PGL, also known as glomus tympanicum, is the most common primary tumor of the middle ear. The terms glomus tympanicum and tympanomastoid glomus tumors are reserved for lesions that arise from the middle ear without the involvement of the jugular bulb [3,4]. These are highly vascular tumors that arise from neural crest-driven glomus bodies along the course of Jacobson’s nerve and Arnold’s nerve [4,7].

Tympanomastoid PGL can be confined to the middle ear at the time of presentation. However, it is not uncommon to encounter tumors that have grown out of the confines of the middle ear, occupying the mastoid cavity, eustachian tube, and hypotympanic cells or protruding towards the external ear canal. In more advanced cases, carotid canal involvement might also be evident [3,8]. 

Over the years, various classification systems of tympanomastoid PGL have been proposed by different scientists. However, the two most widely accepted classification schemes are the ones developed by Fisch and Mattox (Table 1) [9] and Glassock and Jackson [10].

Fisch and Mattox classified tympanomastoid PGLs into A and B based on the radiologic extent of the lesion. According to this classification, class A tumors are confined to the middle ear cleft, whereas class B tumors go beyond the confines of the middle ear cleft to involve different tympanomastoid regions such as the hypotympanum, mastoid air cell system, and carotid canal [11]. 

We adopted the Fisch and Mattox classification system and added subclassifications based on clinical findings and involvement of the hypotympanum, sinus tympani, facial nerve, and carotid canal (Table 2). Furthermore, we developed a management algorithm based on this subclassification [3,4,12]. 

A comprehensive clinical assessment is crucial for accurately diagnosing tympanomastoid PGL, which shows a female predominance and is common between the ages of 20 and 60 [4,13]. The most common presenting symptoms reported are pulsatile tinnitus, hearing loss, and aural fullness [3,7,8,13,14,15]. Conductive hearing loss is the most common audiologic pattern, whereas the degree of loss of bone conduction depends on the extent of cochlear invasion [16]. 

It is extremely unusual to discover functional secreting tympanomastoid PGLs. If a patient has symptoms that indicate elevated catecholamine levels, such as episodic hypertension, palpitation, headache, flushing, or diarrhea, it is recommended to determine the catecholamine level and search for a second tumor in the neck, thorax, or abdomen using the recommended radiologic modalities [17,18]. Functional tumors should be evaluated and managed by endocrinologists before surgery [7,13,16,19]. 

Genetic testing, on the other hand, is a valuable diagnostic method for detecting lesions at an early stage and determining metastatic potentials. According to the Endocrine Society and European Society of Endocrinology, it is recommended that all head and neck paraganglioma patients undergo screening for *SDHx* and SDHB pathogenic variants since 40% of head and neck PGL patients have germline mutation and 25% of patients with SDHB pathogenic variants have metastasis [13,20,21,22,23,24,25,26]. 

Although it is extremely rare, malignancies can still occur in patients with head and neck PGL. However, to date, there are no molecular, cellular, or histopathologic criteria that can be used to define the presence of metastatic lesions in patients with head and neck PGL [21,27]. The mere presence of paraganglionic tissue in areas where it is not normally found may suggest the presence of metastatic lesions. However, the diagnosis of metastasis is confirmed by histopathologic examination [20,21,27]. Cervical lymph nodes are the most common area of metastasis [20,28], but head and neck PGL can still spread to distant organs such as the lung, bone, and liver [13,20,27]. The significance of metastatic nodes and their prognostic implication is yet to be further studied [28]; however, certain factors such as SDHB pathogenic variant, positive family history of paraganglioma, younger age at presentation, functional secretory tumors and rapidly increasing size are factors associated with a higher risk of metastasis [13,22,24,25,26,29]. 

**Figure 1:**
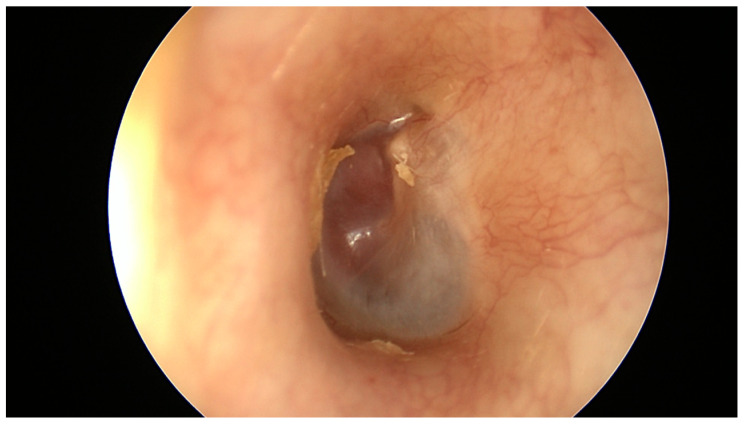
Otoscopic examination showing retrotympanic mass.

Upon otoscopic examination, tympanomastoid PGL generally appears as a red retrotympanic mass [3,13,30] (Figure 1). Brown’s sign, tumor blanching with pneumatic otoscopy can be seen in half of the cases [12]. However, larger tumors may have overlapping presenting symptoms with tympanojugular PGL, which demands thorough preoperative clinical and radiological evaluations.

**Figure 2:**
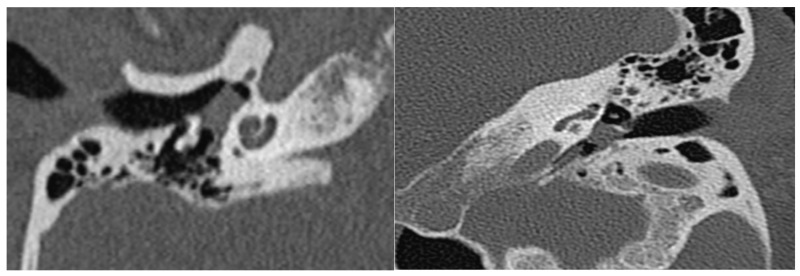
Coronal and axial HRCT sections showing a soft tissue density in the middle ear cavity.

**Figure 3:**
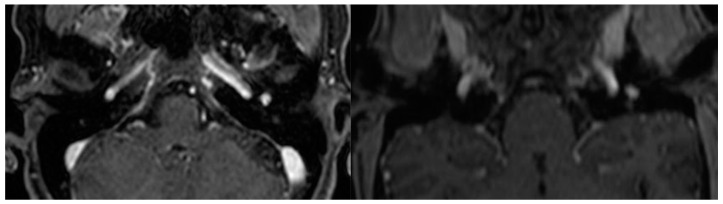
Axial and coronal MRI with gadolinium enhancement sections showing avidly enhancing mass in the middle ear cavity.

The use of HRCT as an initial modality of investigation helps determine the extent of the lesion, its relation with structures that traverse the middle ear cavity, and the status of the ossicular chain, cochlea, fallopian and carotid canals, and jugular plate. A soft tissue density around the promontory is frequently observed in HRCT (Figure 2). Whereas T1 hypointensity, T2 hyperintensity, and avid contrast enhancement are typical MRI characteristics of tympanomastoid PGL (Figure 3) [3,15,25,26,31]. 

**Figure 4:**
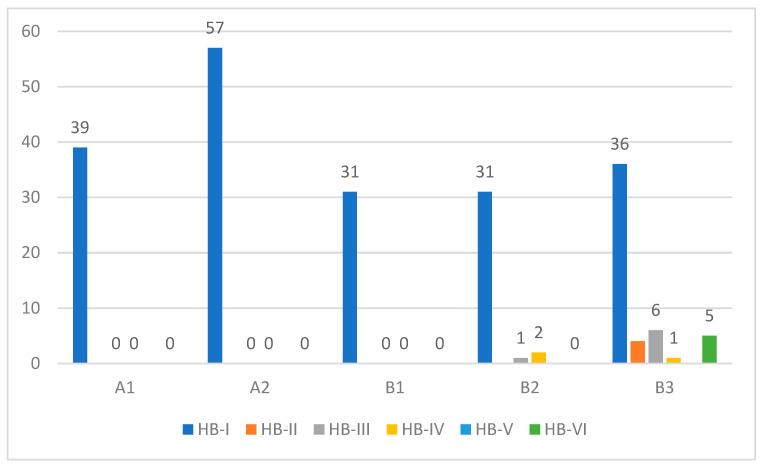
Preoperative facial nerve function (HB grading system) for each class of TMP.

Currently, radiologic advancements, along with well-developed surgical management algorithms (Figure 4) and refined techniques, make proper tumor staging and complete tumor removal possible [4,15,30,31]. 

Nowadays complication and recurrence rates as well as long-term facial function and hearing outcomes are better when compared with other surgically treatable middle ear conditions [4,8]. 

In this article, we present the long-term outcomes of surgically treated paraganglioma A and B cases at our center for over 40 years, which, to the best of our knowledge, is the largest series in the English-language literature.

## 2. Materials and Methods

### 2.1. Objective and Study Design

1. To demonstrate the overall long-term outcome of surgically treated tympanomastoid PGLs.
2. To analyze long-term facial function outcomes in surgically treated tympanomastoid PGLs.
3. To depict the preoperative and postoperative hearing patterns among different types of tympanomastoid PGLs.
4. To determine the rate of recurrent tumors.

Retrospective medical record review.

### 2.2. Methods

Medical records of patients with surgically managed class A and B tympanomastoid PGLs between the years 1983 and 2023 were thoroughly evaluated. All patients who underwent surgery for class A-B tympanomastoid PGLs and had complete medical records were included in the study. Clinical, audiologic, and radiological data were collected for all cases included in this study. Facial nerve function was graded using the House–Brackmann (HB) grading system [32] both pre and postoperatively. Tumor classification was made using modified Fisch and Mattox classification (Table 3). All patients had preoperative contrast-enhanced HRCT scans and MRIs to determine the extent of the lesion and exclude the involvement of the jugular bulb. The surgical approach used for each patient was based on an algorithm developed for each class and subclass of tumors [30] (Figure 1). This algorithm is practically based on the principle of offering adequate surgical exposure according to the location and extent of the tumor. Postoperative follow-up constitutes serial clinical, audiometric, and radiologic elements from the time of surgical intervention to the last outpatient clinic visit.

All cases were operated either by the senior otoneurosurgeons of the group or under their supervision. Small class A1 tumors were managed with the standard transcanal procedure as in stapes surgery, whereas A2 cases with further anterior extension were treated with wider access using the retroauricular-transcanal approach. All class B tumors required a transmastoid approach. Class B1 cases were managed with canal wall up mastoidectomy with a facial recess (posterior tympanotomy) approach aiming to control the most posterior and medial limit of the tumor. This approach can be extended distally by sacrificing the chorda tympani nerve to gain more access to control the hypotympanic area. Class B2 tumors can be managed with canal wall up mastoidectomy with posterior and subfacial tympanotomy. Finally, class B3 cases, with more extensive tumors eroding the carotid canal, are managed with subtotal petrosectomy (SP) with middle ear obliteration. 

Data were processed and analyzed using IBM SPSS Statistics (Version 27) software. Descriptive analyses were carried out by calculating the number and percent for categorical variables and the mean and range for continuous variables. Statistical significance was accepted at *p*-value < 0.05. The Ethical Committee of Casa Di Cura Hospital, Piacenza, Italy, approved this study.

## 3. Results

### 3.1. Demographic Data

Out of a total of more than 450 temporal bone PGLs operated at Gruppo Otologico, we studied a total of 219 tympanomastoid paraganglioma class A and B cases, which were surgically treated in our center. All cases were operated either by the senior otoneurosurgeons of the group or under their supervision using similar surgical equipment. 

Females made up 86% (*n* = 183) of total patients operated on while the rest, 14% (*n* = 30), were males, making the female/male ratio 6:1. Mean age at the time of diagnosis was 54 years old with the range from 5 years old to 87 years old. 

### 3.2. Clinical Features

Duration of symptoms before initial presentation ranged from one month to 30 years, with the mean duration of illness being 33 months.

The most common symptoms at presentation were hearing loss (80%), pulsatile tinnitus (77%), and vertigo (15%). Total hearing loss (anacusis) was present in 15 (7%) of patients, while complete facial paralysis at presentation was seen in five (2%) of patients, and all cases had class B tympanomastoid PGL. Finally, there were different degrees of facial nerve paresis in 14 (6%) cases (Figure 1). 

Initial otoscopic examination revealed a retrotympanic mass in all patients. Two patients with class A1 tympanomastoid PGL have a dry central perforation. Furthermore, 10 (5%) patients had otorrhea and otalgia at the time of their first otoscopic evaluation. 

A total of 12 patients (6%) were surgically treated for the same complaint elsewhere, while three patients received radiation therapy before their presentation to our center.

### 3.3. Classification and Surgical Management

**Figure 5:**
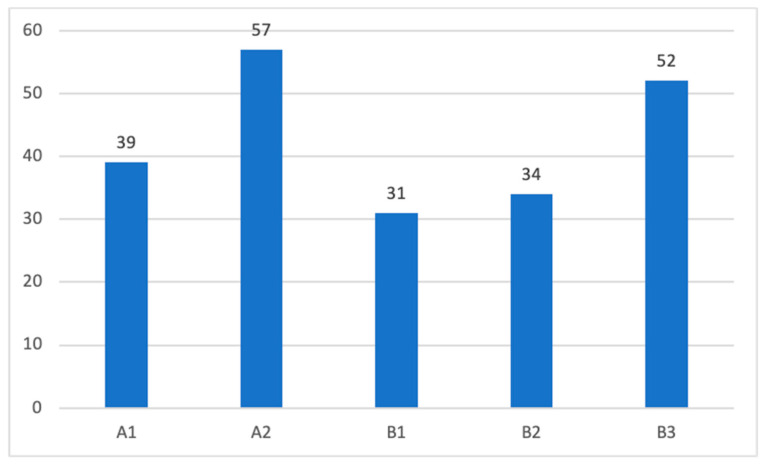
Distribution of patients according to the modified Fisch–Mattox classification scheme.

Among the total surgically treated tympanomastoid PGL cases at our center from 1980 to 2023, 45% were class A, and 55% were class B cases. Patients were distributed for each class according to the modified Fisch and Mattox classification system (Figure 5). 

**Figure 6:**
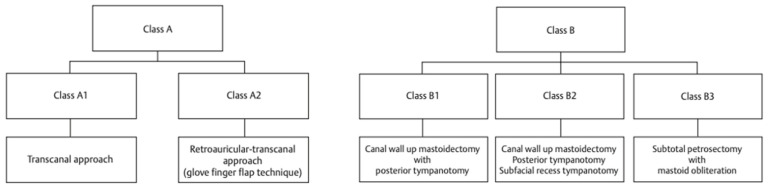
Algorithm for surgical management of tympanomastoid PGLs [30].

The goal of surgical treatment in tympanomastoid PGL is gross total removal. The treatment algorithm [30] we have developed for the choice of the appropriate surgical approach is shown in Figure 6.

All class A1 tympanomastoid PGLs were excised via transcanal (31%) and retroauricular-transcanal (69%) approaches, whereas the majority of class A2 (89%) were removed via retroauricular-transcanal approach.

For class B1 tympanomastoid PGL tumors, canal wall up mastoidectomy with posterior tympanotomy was performed in all cases except two where it was necessary to perform canal wall down (*n* = 1) and subtotal petrosectomy with middle ear obliteration (*n* = 1) to achieve complete surgical excision. 

The entire class B2 tympanomastoid PGL cases were managed using canal wall down tympanoplasty SP with middle ear obliteration.

**Figure 7:**
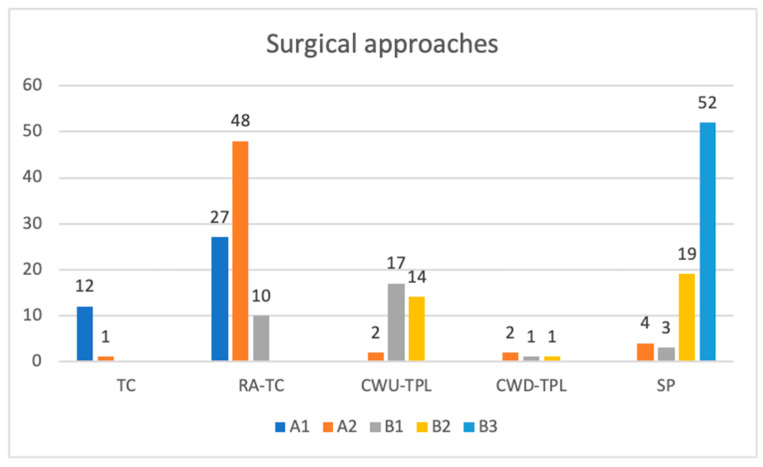
Surgical approaches. TC—transcanal; RA-TC—retroauricular-transcanal; CWU-TPL—canal wall up tympanoplasty; CWD-TPL—canal wall down tympanoplasty; SP—subtotal petrosectomy.

Ninety-eight percent of class B3 cases were managed with SP, whereas it was necessary to perform simultaneous SP with primary facial nerve resection and anastomosis in one case and infratemporal fossa type A (IFTA) in another. The overall distribution of all surgical approaches we used at our center to achieve complete gross tumor removal is shown in Figure 7.

Total surgical excision was achieved in 199 (93%) of our cases whereas near-total tumor removal was performed in the rest of the cases which were all class B tympanomastoid PGLs (B3 = 11, B2 = 3). 

Concomitant cholesterol granuloma was found in two class B1 cases and a single class B2 case presented with simultaneous middle ear cholesteatoma with ossicular chain erosion. A second-stage procedure with the aim of audiological rehabilitation using a total reconstruction prosthesis (TORP) was performed in all of these cases. 

Ossicular chain preservation was achieved during surgery in 95% (*n* = 128) of cases who were treated in all surgical approaches except SP. Furthermore, two out of six patients with ossicular chain removal underwent second-stage ossiculoplasty. 

Cochlear erosion was present in two class B3 tympanomastoid PGL cases. Both of these cases had preoperative anacusis and were managed with complete tumor removal via an SP approach with simultaneous cochlear implantation.

Intraoperative cerebrospinal fluid (CSF) leak was evident in eight cases (B3 = 7 and B2 = 1) without postoperative complication during subsequent follow-ups. Moreover, there were four cases with meningoencephalic hernia who underwent SP for class B3 tumors. Finally, there was not a single case of postoperative wound infection. 

### 3.4. Hearing Outcome

Audiometric outcomes were assessed by comparing the air-bone gap (ABG) of the preoperative and postoperative results for class A, B1, and B2 tympanomastoid PGL patients treated with transcanal and retroauricular approaches. However, for class B3 cases, we compared only the BC results because almost all cases were managed with SP with middle ear obliteration. 

**Figure 8:**
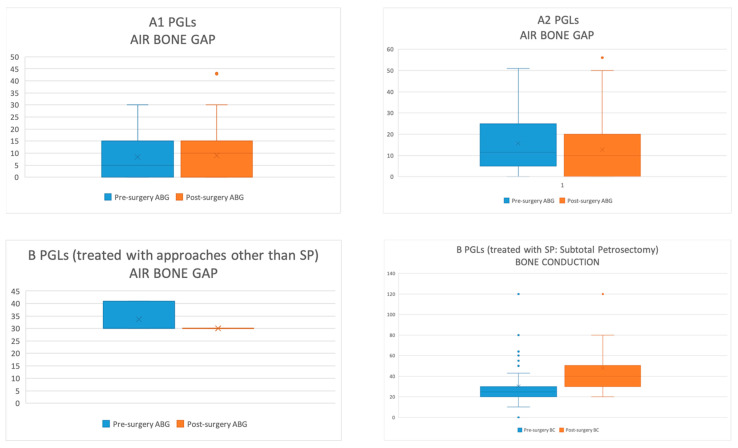
Preoperative and postoperative audiological results for class A and B tympanomastoid PGLs.

For class A1, A2, B1, and B2 tympanomastoid PGLs, there was an obvious improvement of postoperative AC and ABG with a slight increment in BC which could be attributable to surgical manipulation. Details of audiologic outcomes are beyond the scope of this article and will be discussed in detail in our subsequent work. Thus, the graphic comparison of pre-operative and post-operative audiological outcomes is summarized in Figure 8.

### 3.5. Facial Nerve Outcome

**Figure 9:**
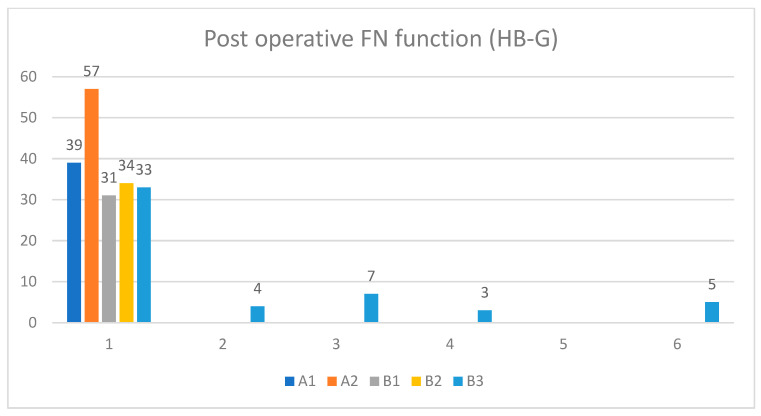
Postoperative facial nerve function (HB grading system).

Preoperative facial nerve paresis was evident in 14 class A and B tumors ranging from HB-II to HB-IV. In contrast, 5 class B3 patients had complete facial nerve paralysis (HB-VI) at presentation. Intraoperative facial nerve status includes either simple erosion of the fallopian canal (*n* = 12) or gross tumor infiltration (*n* = 10). All patients who had gross facial nerve infiltration had facial nerve paresis/paralysis at the initial presentation. The most common site of facial nerve involvement was the tympanic segment, followed by the second genu, mastoid segment, and geniculate ganglion. In patients with gross infiltration, a segment of the involved facial nerve was removed, and reconstruction was performed. Only one case underwent a sural nerve graft, whereas primary end-to-end anastomosis was performed in the rest of the cases, with excellent outcomes at one-year follow-up. Postoperatively, facial nerve deficit was apparent only in class B2 and B3 tumors (Figure 9), and this finding has a strong correlation with preoperative facial nerve function as well as intraoperative findings (*p* < 0.05).

### 3.6. Follow-Up and Complications 

The clinical and neuroradiologic follow-up time ranged from 1 month to 26 years, with a mean follow-up of 4 years. The most common late complications include permanent tympanic membrane perforation (7%), impaired facial nerve function (7%), and recurrence (3%). Minor complications like transient vertigo, external ear canal stenosis, external ear canal cholesteatoma pearl, and worsening of tinnitus occurred in 6% of all cases (Table 3).

**Table 3:** Postoperative complications.

Late Complications	Percentile % (*n*)
Permanent TM perforation	7% (14)
Cholesteatoma	1% (3)
Worsening of tinnitus	1% (3)
Recurrence	3% (6)
Facial nerve paralysis	7% (15)
Others	6% (12)

Recurrence occurred in seven (3%) patients, with the mean duration of recurrence being 98 months from the date of surgery. Four of these cases were class A, and the rest were class B. All except one case underwent revision surgery via retroauricular approach, whereas a single class B3 case was treated with IFTA. 

## 4. Discussion

Tympanomastoid PGL is the most common middle ear pathology. Complete surgical excision with excellent postoperative facial function, hearing, and overall outcome is possible due to the advancement of radiologic diagnostic tools and refinement of surgical management options [3,4,7]. 

### 4.1. Clinical Features

Females are five to six times more affected than males [4,33,34]. The most common presenting symptoms include pulsatile tinnitus, hearing loss, and otalgia, whereas the most common clinical finding is retrotympanc mass, which is evident during initial otoscopic examination [3,4]. Conductive hearing loss is the most common audiometric finding, even though patients might also have sensory neural hearing loss depending on the extent of cochlear invasion [7,8]. Moreover, a ‘rising sun’ finding during otoscopic examination is suggestive of a mass originating from the hypotympanic area and requires further investigation [7]. Among a series of studies conducted at our center and elsewhere, one was able to demonstrate the increased risk of tympanic membrane perforation with higher classes of tympanomastoid PGL [4]. However, regardless of the status of the tympanic membrane, it should be noted that a biopsy of the mass is not advised [7].

Once we have the diagnosis of tympanomastoid PGL in our mind, the next step should be ruling out the most important differential diagnosis such as inflammatory polyps, cholesterol granulomas, hemangioma, high jugular bulb, aberrant internal carotid artery, tympanojugular PGL, and other malignant middle ear neoplasms such as facial nerve neuroma using HRCT, the initial preferred method of investigation [3,7,8,35]. 

The use of HRCT as an initial modality of investigation helps determine the extent of the lesion, its relation with structures that traverse the middle ear cavity, and the status of the ossicular chain, cochlea, fallopian canal and carotid canals and jugular plate [3,15]. The presence of either bone or air between the mass and the jugular bulb is highly suggestive of the mass arising from the middle ear [3,4,12]. 

Moreover, it is very crucial to differentiate between tympanomastoid and tympanojugular PGLs during the initial evaluation of the HRCT due to differences in preoperative management as well as surgical approaches [3,4,15,35]. It is very important to have a preoperative MRI with gadolinium enhancement if the tumor is in the hypotympanic area and appears to be near the jugular bulb [4]. However, angiography and neuroendocrine tests are not part of routine preoperative examinations in patients with tympanomastoid PGL [7].

### 4.2. Classification and Algorithm of Surgical Treatment

The choice of appropriate surgical approach in the management of tympanomastoid PGL is dictated by the location and extent of the tumor and the status of the intrapetrous carotid canal [3,4]. To classify the tumors preoperatively, the Fisch and Mattox classification [9] is adopted, and further subclassifications (A1, A2, B1, B2, and B3) are added to it [3,4,12]. Apart from the description of the extent of the tumor, it also helps choose the appropriate surgical approach, even highlighting whether the carotid is involved or not and is not entirely based on radiologic findings [4]. We depicted the surgical algorithm we developed based on the modified Fisch and Mattox classification in Figure 3. A detailed description of the specified surgical procedures is thoroughly described elsewhere [3]. Methods that allow us good hearing conservation can be employed for almost all class A and half of class B cases [4]. Whereas, in the rest of the advanced class B cases, factors such as cochlear invasion, inadequate tumor exposure, and the choice of surgical approach, i.e., SP make bone conduction conservation possible during the stage of tumor removal [3,4,30]. 

For class A1 tumors, where the tumor is confined to the middle ear cavity with visible circumferential boundaries, the transcanal approach can be safely used. Class A2 tumors with posterior mesotympanic extension can safely be managed with a retroauricular-transcanal approach [3,30]. 

Moreover, we use the glove finger flap approach we developed in our center by modifying the classic retroauricular-transcanal approach. The hallmark of this approach is adequate canaloplasty and visualization of the entire tympanic ring to gain more access to the surgical field and expose the tumor widely through the ear canal. This approach also provides better control of the ossicular chain and visualization of the oval and round windows and fallopian canal during tumor removal. Furthermore, it makes the use of bipolar electrocautery tumor removal easy while avoiding trauma to the meatal skin and the tympanic membrane [3,4,30]. 

In class B1, where posterior tumor extension is limited to the facial recces area, a canal wall up mastoidectomy with posterior tympanotomy is usually adequate to remove the tumor completely [3,4,30]. 

Whereas, in class B2 tumors where there is an inferior hypotympanic extension, posterior tympanotomy alone is insufficient to expose the entire tumor. Thus, canal wall up mastoidectomy with an extended facial recess approach with further inferior hypotympanic extension, and subfacial tympanotomy represents the appropriate surgical approach [3,4,8,30]. 

In larger class B3 tumors there is involvement of the carotid canal. Another study conducted in our center in 2015 showed a 17% risk of carotid canal involvement [4]. In such cases, SP with middle ear obliteration is the preferred surgical approach. Hearing rehabilitation using different techniques such as bone-anchored hearing aid (BAHA) [36] and middle ear implant devices such as Vibrant Soundbridge [37] can be planned as a second stage procedure [3,4,30].

In cases where the involvement of the jugular bulb is discovered intraoperatively, it is highly recommended either to perform careful tumor dissection or to terminate the procedure and plan IFTA after obtaining appropriately informed consent [3,4,30]. 

We used conventional surgical instruments, including bipolar electrocautery, for the safe excision of paraganglioma tumors from the middle ear in all of our patients. However, the use of different types of lasers, such as Diode Laser, KTP Laser, and Nd-YAG Laser for excision, has been investigated in different works of the literature as an alternative to conventional surgical instruments. These new methods provide complete tumor excision with minimal bleeding [11,38,39]. However, the risk of cochlear damage should be underlined and investigated in further studies aiming at the evaluation of hearing outcomes after the use of lasers in the middle ear [3,4,30]. 

### 4.3. Outcome and Complications

When compared with other middle ear pathologies, the rate of postoperative complication in patients with class A and B tympanomastoid PGLs is comparable and minimal [4,8]. Complete surgical removal of class A and B tympanomastoid PGLs ranged from 94 to 100%, whereas the recurrence rate is between 0 and 5% [3,4,8,15,40,41]. Following the anatomical landmarks while performing mastoidectomy, identification of the facial nerve before starting tumor dissection, and applying bloodless tumor dissection techniques are the cornerstones of excellent postoperative facial nerve outcomes. In our experience, we achieved complete tumor removal in 97% of our cases, and our recurrence rate was 3%, with the mean duration of time between the surgery and recurrence being 98 months. Based on this finding, we recommend long-term postoperative follow-up.

### 4.4. Radiation Therapy (RT)

Based on the excellent postoperative outcomes documented in our 40-plus years of experience, we strongly recommend surgical removal as the primary modality of treatment with a high success rate of complete disease removal and less morbidity [3,4,35]. Numerous studies are in line with our recommendations [8,16,41,42]. Thus, we believe it is appropriate to consider the radioresistant nature of these tumors and RT-associated complications such as external ear canal stenosis, RT-induced neoplasms, and osteoradionecrosis before exposing patients to radiation therapy [3,4]. However, RT is still advocated as the initial modality of treatment by others [16,25,43,44]. Thus, if there is still a place for RT as a primary mode of treatment, it should be in selected cases where surgery is strongly contraindicated [3,4,16]. 

## 5. Conclusions

Tympanomastoid PGLs represent the most common primary neoplasm of the middle ear. The most common presenting symptoms include hearing loss, tinnitus, and impaired facial nerve function, whereas the presence of retrotympanic mass was evident in all cases at the time of initial otoscopic evaluation. These particular symptoms should not be overlooked during office examinations. Proper documentation of facial function and audiometric evaluations are crucial elements of preoperative workup. Office biopsy of the mass is ill-advised as there is a risk of massive bleeding. The most preferred preoperative radiological examination is HRCT, whereas MRI with or without gadolinium enhancement is reserved for cases with a dilemma of the carotid artery or jugular bulb involvement. Moreover, a total body scan is warranted when there are catecholamine-induced symptoms and suspicion of metastatic lesions. SDHB pathogenic variant, positive family history of paraganglioma, younger age at presentation, functional secretory tumors, and rapidly increasing size are factors associated with a higher risk of metastasis. Currently, it is recommended that all head and neck PGL patients undergo screening for *SDHx* and SDHB pathogenic variants since 40% of head and neck PGL patients have germline mutation and 25% of patients with SDHB pathogenic variants have metastatic lesions. The main goal of tympanomastoid PGL treatment is complete disease removal with hearing and facial function preservation. Surgical treatment remains the preferred modality with the benefits of complete disease removal, lower rate of recurrence and complication, and acceptable postoperative hearing level.

## Figures and Tables

**Table 1:** Fisch and Mattox classification of the middle ear and mastoid glumus tumors [9].

Type	Description
A	Tumors limited to the middle ear cleft
B	Tumors limited to tympanomastoid compartment of the temporal bone

**Table 2:** Modified Fisch and Mattox classification of middle ear and mastoid glomus tumors [3].

Class	Description
A	Tumors limited entirely to the middle ear cleft
A1	Tumors completely visible on otoscopic examination
A2	Tumor margins are not visible on otoscopy. Tumor may extend anteriorly to the Eustachian tube and/or to the posterior mesotympanum
B	Tumors limited to the tympanomastoid compartment of the temporal bone
B1	Tumor filling the middle ear cleft with extension into the hypotympanum and tympanic sinus
B2	Tumor filling the middle ear cleft, extending into the mastoid and medially to the mastoid segment of the facial nerve
B3	Tumor filling the middle ear cleft, extending into the mastoid with erosion of carotid canal

## Data Availability

The data presented in this study are available in this article.
